# Validation of DM-Scan, a computer-assisted tool to assess mammographic density in full-field digital mammograms

**DOI:** 10.1186/2193-1801-2-242

**Published:** 2013-05-24

**Authors:** Marina Pollán, Rafael Llobet, Josefa Miranda-García, Joaquín Antón, María Casals, Inmaculada Martínez, Carmen Palop, Francisco Ruiz-Perales, Carmen Sánchez-Contador, Carmen Vidal, Beatriz Pérez-Gómez, Dolores Salas-Trejo

**Affiliations:** National Center for Epidemiology, Carlos III Institute of Health, Monforte de Lemos 5, Madrid, 28029 Spain; Consortium for Biomedical Research in Epidemiology and Public Health (CIBER en Epidemiología y Salud Pública - CIBERESP), Carlos III Institute of Health, Monforte de Lemos 5, Madrid, 28029 Spain; Instituto Tecnológico de Informática, Universidad Politécnica de Valencia, Valencia, Spain; Valencian Breast Cancer Screening Program, General Directorate of Public Health, Valencia, Spain; Centro Superior de Investigación en Salud Pública CSISP, FISABIO, Valencia, Spain; Balearic Islands Breast Cancer Screening Program, Health Promotion for Women and Children, General Directorate of Public Health & Participation, Regional Authority for Health & Consumer Affairs, Balearic Islands, Spain; Cancer Prevention and Control Unit, Catalonian Institute of Oncology (Institut Català d’Oncologia-ICO), Barcelona, Spain; Centro Nacional de Epidemiología, Instituto de Salud Carlos III, Monforte de Lemos 5, Madrid, 28029 Spain

**Keywords:** Mammographic density, Breast density, Density assessment, Computer-assisted tool, Reliability

## Abstract

We developed a semi-automated tool to assess mammographic density (MD), a phenotype risk marker for breast cancer (BC), in full-field digital images and evaluated its performance testing its reproducibility, comparing our MD estimates with those obtained by visual inspection and using Cumulus, verifying their association with factors that influence MD, and studying the association between MD measures and subsequent BC risk.

Three radiologists assessed MD using DM-Scan, the new tool, on 655 processed images (craniocaudal view) obtained in two screening centers. Reproducibility was explored computing pair-wise concordance correlation coefficients (CCC). The agreement between DM-Scan estimates and visual assessment (semi-quantitative scale, 6 categories) was quantified computing weighted kappa statistics (quadratic weights). DM-Scan and Cumulus readings were compared using CCC. Variation of DM-Scan measures by age, body mass index (BMI) and other MD modifiers was tested in regression mixed models with mammographic device as a random-effect term.

The association between DM-Scan measures and subsequent BC was estimated in a case–control study. All BC cases in screening attendants (2007–2010) at a center with full-field digital mammography were matched by age and screening year with healthy controls (127 pairs). DM-Scan was used to blindly assess MD in available mammograms (112 cases/119 controls). Unconditional logistic models were fitted, including age, menopausal status and BMI as confounders.

DM-Scan estimates were very reliable (pairwise CCC: 0.921, 0.928 and 0.916). They showed a reasonable agreement with visual MD assessment (weighted kappa ranging 0.79-0.81). DM-Scan and Cumulus measures were highly concordant (CCC ranging 0.80-0.84), but ours tended to be higher (4%-5% on average). As expected, DM-Scan estimates varied with age, BMI, parity and family history of BC. Finally, DM-Scan measures were significantly associated with BC (p-trend=0.005). Taking MD<7% as reference, OR per categories of MD were: OR_7%-17%_=1.32 (95% CI=0.59-2.99), OR_17%-28%_=2.28 (95% CI=1.03-5.04) and OR_>=29%_=3.10 (95% CI=1.35-7.14). Our results confirm that DM-Scan is a reliable tool to assess MD in full-field digital mammograms.

**Electronic supplementary material:**

The online version of this article (doi:10.1186/2193-1801-2-242) contains supplementary material, which is available to authorized users.

## Introduction

Mammographic density (MD), a strong risk factor for breast cancer, is increasingly used as a phenotype risk marker in clinical, genetic and epidemiological studies (Boyd et al. 2011). Recently, MD has also been proposed as a key feature to tailor screening algorithms according to individual breast cancer risk (Schousboe et al. 2011;Evans et al. 2012).

Different methods for assessing density have been used (Yaffe 2008;Assi et al. 2012). The first qualitative scales that took into account parenchymal patterns have been largely replaced by a quantitative approach that considers percentage of density (PD), or the percentage of the total breast area occupied by dense tissue. Visual inspection has allowed to classify mammograms in semi-quantitative scales with 5, 6 or even 21 categories of PD (Garrido-Estepa et al. 2010;Cuzick et al. 2011). However, achieving high reproducibility and reliability of visual assessment is always a challenge. In order to reduce subjectivity, several computer-assisted methods have been developed. One of such methods, Cumulus, has become the gold standard of quantitative PD assessment, and has shown a similar ability to predict breast cancer compared to visual assessment (Byng et al. 1998). At present, computer-assisted methods were developed for film images and have not been validated with digital mammograms. A comparative study using Cumulus in both types of images concluded that Cumulus underestimates PD in digital mammograms (Harvey 2004). This phenomenon is related with a better recognition of the skin line in digital images which implies the inclusion of more subcutaneous fat under the total area of the breast and a decrease in the relative amount of dense tissue (Harvey 2004).

In many countries, analog mammography is increasingly replaced by digital devices, due to their better performance. This trend has also been observed in Spain, a country with fully established population-based breast cancer screening programs (Ascunce et al. 2010). A recent study shows that the introduction of digital mammography has reduced the rate of false-positive results in Spanish screening programs (Sala et al. 2011).

In this paper, we present DM-Scan, a new semi-automatic tool to measure PD specially developed for digital images. Three different radiologists estimated PD using DM-Scan in a set of digital mammograms already collected in the study DDM-Spain (Determinantes de la Densidad Mamográfica en España – Determinats of Mammographic Density in Spain) (Cabanes et al. 2011). Density estimates using DM-Scan were compared to those previously obtained by visual inspection and with Cumulus estimates. Finally, the discriminative value of DM-Scan was checked testing the association between PD and breast cancer in a case–control study. For this purpose, images from women who subsequently developed breast cancer after screening were collected and compared to those obtained in healthy screened women of a similar age.

## Material and methods

### Development of DM-scan

**Figure 1:**
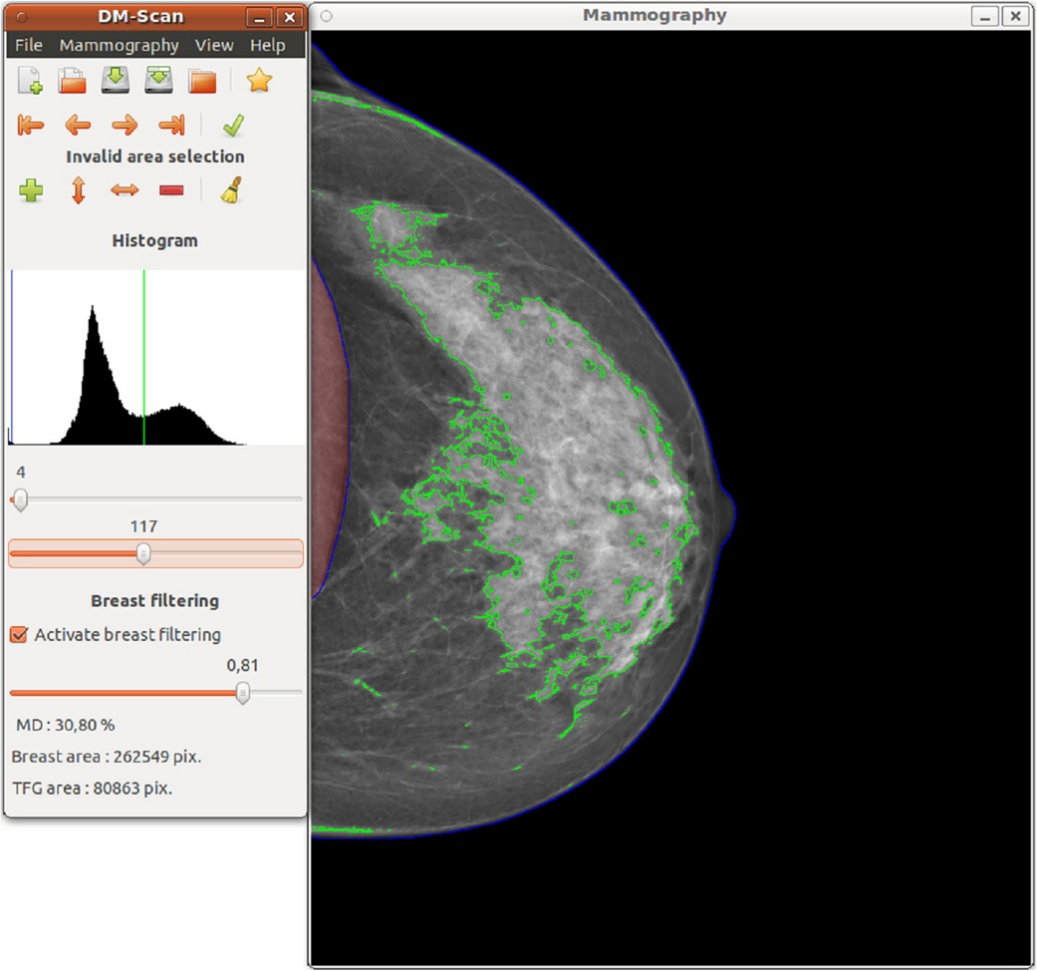
**Interface of DM-Scan, the new semiautomatic tool to assess mammographic density.**

DM-Scan (Figure 1) is a computer-assisted tool aimed at PD assessment in a continuous scale. It has been developed to run both on Windows and Linux operating systems. Given a digital mammogram, this tool identifies pixels belonging to background, fat tissue (FT) and dense tissue (DT) by means of the establishment of two thresholds, called T1 and T2. Then, PD is measured as the relation between the amount of DT and the size of the breast, i.e., PD = DT / (DT + FT) 100. Following, this process is explained in detail.

Firstly, a pre-process is applied to condition the image before tissue segmentation is performed. Three main operations are carried out at this phase: a) contrast and brightness normalization, b) brightness correction according to breast thickness and c) segmentation of the breast and removal of regions of no-interest.

#### Contrast and brightness normalization

To ensure that brightness values depend as much as possible on tissue density and not on other factors related with the acquisition process, a contrast and brightness normalization is desirable. Assuming that minimum and maximum tissue densities are always present on a mammography (subcutaneous fat and connective tissue respectively), minimum and maximum gray-level values should also appear in the histogram. Based on this idea, a histogram stretching operation can be set to normalize brightness and contrast. Options to manually modify brightness and contrast are also available.

#### Brightness correction

X-ray attenuation depends not only on the density of the irradiated tissue, but also on its thickness. The thicker the tissue irradiated, the greater the attenuation and, consequently, the brighter the image. When the mammogram is taken, the breast is compressed between two parallel flat plates, which causes the breast to spread out and have a similar thickness along the plates. However, towards the edge of the breast, the thickness gradually decreases. This is a drawback when the goal is segmenting dense tissue, since thicker regions may look as dense tissue and vice versa. In order to avoid this problem, after estimating breast thickness (Highnam et al. 1998), a brightness correction coefficient k_i,j_ has been applied to each pixel p_i,j_ according to a user-defined parameter α Є [0:1] as specified below:

$$K_{i,j}=\alpha +\left(1-\alpha \right)d_{i,j}$$

where d_i,j_ is the horizontal distance from p_i,j_ to either the internal border of the image (craniocaudal view) or the pectoral muscle if present (mediolateral oblique) divided by the total distance between this border and the breast edge at row i, i.e., di,j = 0 when pi,j coincides to the border of the image, and di,j = 1 when p_i,j_ coincides with the edge breast. Values of α = 1 leaves the image unchanged, while values of α < 1 attenuates the brightness as we approach to the internal part of the mammogram. A lower α corresponds to a greater attenuation.

#### Breast segmentation and removal of unwanted regions

Usually, mammograms contain other objects besides the breast, such as labels and/or the pectoral muscle. Breast segmentation is semi-automatically performed by finding a threshold value T1 that discriminates between background and object pixels. The biggest object found is considered to be the breast, while the remainders are considered regions of non-interest and, therefore, removed. Nevertheless, this process cannot discard objects connected to the breast. To fix this problem, the user can modify the proposed T1 threshold and also manually invalidate other regions/objects not detected in the previous process.

Once the image has been preprocessed and the breast has been segmented by means of T1, a second threshold T2 must be manually set to separate dense and fat tissue, which allows to measure the dense tissue (DT) and the non-dense or fat tissue (FT). Finally, PD is computed as DT / (DT + FT) 100. Figure 1 presents an example of digital mammogram viewed in the DM-Scan screen.

### Reproducibility of DM-scan, comparison with visual scales (Wolfe, Tabar, BIRADS and Boyd scales) and with cumulus

Digital mammograms used in this project were collected as part of DDM-Spain (Determinants of Density in Mammograms in Spain), a cross-sectional study to investigate the main determinants of high breast density in Spanish women. More than 3500 women aged 45–68 years were recruited at 7 screening centres in Spain. Information regarding lifestyle factors was obtained by trained interviewers at the screening centre. Participants were also weighted and measured using a standardized protocol (Pollan et al. 2012). Mammographic density was visually assessed by a single radiologist on the craniocaudal view of the left breast. The study was approved by the ISCIII ethics committee and informed consent was obtained from all participants.

Mammographic density was visually assessed in the DDM-Spain study by a single radiologist with high intra-rater agreement (Garrido-Estepa et al. 2010). He classified MD according to three qualitative scales (Wolfe, Tabar & BIRADS) and a semiquantitative scale (Boyd) with 6 PD categories (0%, <10%, 10-25%, 25-50% 50-75% and >75%). Qualitative scales measure parenchymal patterns and their categories describe the distribution of the dense tissue inside the breast. It has been reported that Wolfe’s categories N1, P1, P2 and DY correspond approximately to Tabar patterns II, III, IV and V (Assi et al. 2012).

In the present study, a set of 655 digital mammograms from women attending the screening centres located in Palma de Mallorca (Balearic Islands) and Barcelona (Catalonia) were selected. Both centres have full-field digital mammography devices (a Hologic-Lorad M-IV in Palma de Mallorca and a Siemmens MAMMOMAT *Novation*^DR^ in Barcelona). Digital mammograms had already been processed and stored in DICOM format. None of these screening centers stored unprocessed (raw) images.

DICOM images were converted to PNG format in order to be read using DM-Scan. Three radiologists with long experience in mammographic reading were trained with the new tool, using a set of digital images that were not part of the present study. After training, they separately assessed PD in the batch of digital mammograms previously described. Finally, the only radiologist with experience using Cumulus read the whole batch of mammograms using this tool.

Reproducibility of PD measures with DM-Scan was estimated by the concordance correlation coefficient (Lin 1989). The pairwise agreement was also visually evaluated plotting Bland and Altman graphics (Bland & Altman 1986).

In order to compare DM-Scan and Cumulus performance with visual scales, we studied the distribution of PD measures per category of the four visual methods (Wolfe, Tabar, BIRADS and Boyd). In addition, we quantified the agreement between the single visual quantitative scale, Boyd scale, and the two computer-assisted methods, DM-Scan and Cumulus, using weighted kappa statistics (quadratic weights). For this purpose, DM-Scan and Cumulus readings were categorized considering the 5 cut-offs proposed by the Boyd scale. Finally, the agreement between DM-Scan and Cumulus was evaluated computing concordance correlation coefficients and drawing the corresponding Bland-Altman graphics.

### Association between several determinants of MD and MD measures using DM-scan and cumulus

The association between several determinants of MD density, such as age, menopausal status, BMI, family history of breast cancer, parity and use of hormonal replacement treatment, with the PD estimators obtained by each radiologist with DM-Scan was tested in a regression mixed model, with PD as the dependent variable and the screening center as a random effect term. The same procedure was used to estimate the association of these variables with PD measures obtained using Cumulus.

### Association of MD and subsequent breast cancer using DM-scan

In order to test the performance of DM-Scan to detect differences in PD in mammograms from healthy women and those who subsequently develop breast cancer, we set-up a case–control study including all breast cancer cases diagnosed in women attending the Burjasot screening center in Valencia, where full-field digital images had been used for more than 4 years (Senographe 2000D Full Field Digital Mammography System). All breast cancer cases diagnosed in women attending screening there between the years 2007 and 2010 were included in this study. For each case, a matched control was randomly chosen among women who were screened the same year and had a similar age (+/− 2 years).

For MD assessment, the left craniocaudal mammogram was selected, when the two views were available, otherwise the mediolateral oblique view of the left breast was used. When the time elapsed between the date of screening and the date of diagnosis was lower than 3 years, the craniocaudal or mediolateral oblique view of the contralateral breast was used. After excluding two cases with breast implants in the contralateral breast, a total of 127 cases and controls were identified. The mammogram was not available for 12 cases and 8 controls, rendering a final sample of 115 cases and 119 controls.

For cases and controls, information on age, menopausal-status and self-reported BMI was extracted from the questionnaires administered by the screening program at the corresponding round.

Unconditional logistic models were used to assess the association between DM-Scan estimates of density and BC risk, allowing for age, menopausal status and BMI as possible confounders. PD was categorized using as cut-offs the quartiles observed in the control group. The possible linear trend was assessed considering PD as a continuous variable. Finally, the discriminative power of DM-Scan readings was computed estimating the area under the receiver operating characteristic curve (AUC), adjusting by age and BMI as covariates. A nonparametric approach was used and 95% confidence intervals were obtained via bootstrap re-sampling (5000 samples).

All statistical analyses were performed using the STATA version 12.0 software program (Stata Corp, College Station, TX).

### Ethical considerations

DDM-Spain study protocol was formally approved by the Institutional Review Board at the Carlos III Institute of Health. Participants signed an informed consent. The case–control study was approved by the CSISP & Valencia General Directorate of Public Health Ethics Committee (*CEIC Dirección General de Salud Pública y Centro Superior de Investigación en Salud Pública*). The breast cancer screening program in Valencia gathers information from all screening participants, all the required data were provided, in an anonymous way, by the screening personnel. Both studies were conducted in compliance with the Helsinki Declaration.

## Results

### Inter-rater agreement using DM-scan

Table 1 presents the average difference in the pair-wise comparison between radiologists’ readings, the 5th and 95th percentiles of the distribution of these differences and the corresponding concordance correlation coefficients. Mean differences in PD were lower than 2%, and almost 90% of the differences between readers were lower than 10%. All concordance correlation coefficients were higher than 0.90. Bland-Altman graphics are provided as Additional file 1.

**Table 1:** **Pairwise comparison of DM-Scan estimates of percentage of density (PD) by the three raters**

	Rater 1		Rater 2	
	Difference	Concordance correlation	Difference	Concordance correlation
	Mean		Mean	
		Coefficient		Coefficient
	(P_05_-P_95_)^a^	(95% CI)^b^	(P_05_-P_95_)^a^	(95% CI)^b^
	PD (Rater1) - PD (Rater2)			
Rater 2	-1.6%	0.921		
	(-10.2% to +6.6%)	(0.910-0.933)		
	PD (Rater1) – PD (Rater3)		PD (Rater2) – PD (Rater3)	
Rater 3	+0.3%	0.928	+1.9%	0.916
	(-7.7% to +9.3%)	(0.917-0.939)	(-5.9% to 11.5%)	(0.904-0.929)

^a^ 5th & 95th percentiles of the differences between PD estimated by these raters.

^b^ 95% Confidence Intervals.

### Comparison of DM-scan, cumulus and visual reading

**Figure 2:**
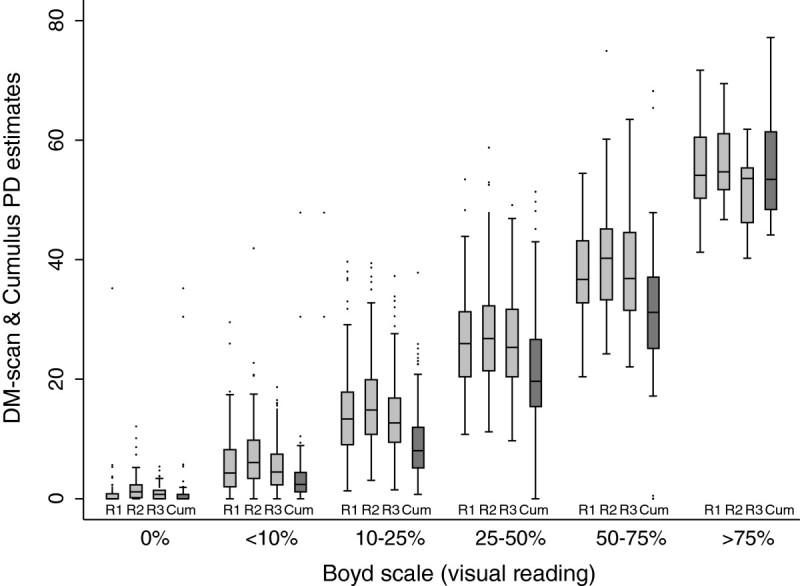
**Distribution of the percentage of density (PD) obtained with DM-Scan (light gray) by three raters (R1 R2 & R3) and Cumulus (dark gray) by one rater (Cum) per categories of PD according to visual assessment (Boyd scale).**

Table 2 shows the distribution of the participants’ mammograms according to the four visual scales and the results obtained using DM-Scan (three raters) and Cumulus (one rater) in each category. For the three qualitative scales, namely Wolfe Tabar and BIRADS, average PD values obtained using computer-assisted methods increased as we move from a category to the next, with the single exception of Tabar scale, for which the average values obtained in the two upper categories were very similar. However, quantitative measures obtained either with DM-Scan or Cumulus showed a high degree of overlapping, something that may be in part explained by the qualitative nature of these scales. Figure 2 and Table 2 shows the distribution of DM-Scan and Cumulus readings per categories of the Boyd scale, which classifies MD according to a visual estimation of PD, together with the corresponding weighted kappa statistis. There was a substantial agreement between PD measured by visual assessment and readings obtained with both, DM-Scan and Cumulus, though the concordance estimate was higher using DM-Scan (kappa statistics ranging 0.789 to 0.805 with DM-Scan against a value of 0.697 with Cumulus).

**Table 2:** **Comparison between mammographic density assessment using different visual scales (1 rater), DM-Scan (3 raters) and Cumulus (1 rater)**

		DM-Scan Rater 1		DM-Scan Rater 2		DM-Scan Rater 3		Cumulus (Rater 1)	
Classification	N	Mean	P_05_-P_95_	Mean	P_05_-P_95_	Mean	P_05_-P_95_	Mean	P_05_-P_95_
**Qualitative scales**									
Wolfe^a^									
N1	43	1.8%	0.0%-5.8%	3.6%	0.0%-12.5%	1.6%	0.0%-6.5%	2.3%	0.0%-6.5%
P1	327	8.2%	0.0%-19.2%	10.4%	1.5%-24.8%	7.9%	0.0%-17.1%	5.4%	0.0%-17.1%
P2	166	25.6%	12.3%-1.3%	26.7%	11.7%-45.8%	25.7%	5.7%-38.7%	19.3%	5.7%-38.7%
DY	101	33.5%	15.2%-55.7%	34.1%	13.8%-60.1%	32.7%	9.3%-61.4%	29.2%	9.3%-61.4%
Tabar^b^									
II	43	1.8%	0.0%-5.8%	3.6%	0.0%-12.5%	1.6%	0.0%-6.5%	2.3%	0.0%-6.5%
III	381	10.1%	0.0%-26.0%	12.2%	1.7%-12.5%	9.8%	1.1%-23.2%	6.8%	0.1%-22.6%
IV	155	30.8%	13.4%-53.2%	31.5%	12.3%-54.7%	30.6%	13.0%-50.5%	24.9%	5.9%-50.9%
V	58	29.4%	12.8%-51.6%	29.8%	10.4%-52.5%	28.7%	12.3%-53.8%	25.6%	9.4%-51.4%
BIRADS density^c^									
1	340	6.8%	0.0%-17.5%	8.8%	0.4%-21.5%	6.5%	0.0%-15.8%	4.6%	0.0%-13.9%
2	183	21.2%	10.8%-36.6%	21.9%	10.1%-38.3%	20.8%	10.2%-34.6%	15.4%	4.2%-30.6%
3	89	34.1%	24.0%-48.3%	35.4%	22.3%-50.4%	33.9%	21.8%-49.2%	28.2%	15.4%-46.6%
4	25	47.6%	30.9%-68.0%	51.3%	32.0%-69.4%	47.6%	32.5%-61.8%	45.4%	16.8%-68.3%
**Semiquantitative scale**									
Boyd categories									
0%	46	1.0%	0.0%-5.3%	2.1%	0.0%-8.7%	1.1%	0.0%-3.8%	1.6%	0.0%-5.8%
<10%	186	5.7%	0.0%-14.3%	7.1%	1.5%-14.5%	5.3%	0.8%-12.3%	3.4%	0.0%-8.2%
10%-25%	195	14.1%	3.8%-27.1%	16.0%	7.0%-28.8%	13.6%	5.1%-23.5%	9.2%	2.4%-20.4%
25-50%	139	26.2%	13.6%-40.2%	27.6%	14.9%-44.1%	26.2%	12.5%-41.5%	21.4%	7.3%-37.4%
50-75%	59	37.5%	28.2%-48.8%	39.5%	27.5%-51.1%	38.0%	26.1%-53.4%	31.4%	0.5%-47.9%
>75%	13	56.0%	41.3%-71.7%	57.0%	46.8%-69.4%	51.6%	40.3%-61.8%	55.0%	44.2%-77.2%
**Agreement Boyd – DM-Scan**								**Agreement Boyd –Cumulus**	
Weighted kappa			0.801		0.789		0.805		0.697
(95% Confidence Interval)			(0.777-0.823)		(0.764-.812)		(0.783-.825)		(0.652-.738)
**Agreement Cumulus – DM-Scan**									
Mean Difference (DM-Scan – Cumulus)		Mean Difference	CCC ^e^	Mean Difference	CCC ^e^	Mean Difference	CCC		
(P_05_-P_95_^d^)		+3.7%	0.841	+5.3%	0.803	+3.5%	0.842		
Concordance Correlation Coefficient (CCC)		(+0.0% to +42.3%)	(0.820-0.863)	(+1.3% to+45.6%)	(0.777-0.828)	(+0.8% to +43.3%)	(0.820-0.864)		
(95% Confidence Interval)									

^a^ Wolfe classification:

N1: Breast composed almost completely of fat, with perhaps just a few fibrous connective tissue strands.

P1: Breast composed mainly of fat, although up to a quarter of the sub-areolar area may show beaded or cord-like areas of ducts.

P2: More severe involvement of the breast, with a prominent duct pattern occupying more than one quarter of breast volume.

DY: Breast typically contains extensive regions of homogeneous mammographic densities. The proportion of density is greater than that of the fat.

^b^ Tabár classification:

I Mammogram composed of scalloped contours with some lucent areas of fatty replacement and 1 mm evenly distributed nodular densities (none of our mammograms were classified in this category).

II Mammogram composed almost entirely of lucent areas of fatty replacement and 1-mm evenly distributed nodular densities.

III: Prominent ducts in the retroareolar area.

IV: Extensive, nodular and linear densities with nodular size larger than normal lobules.

V: Homogeneous ground-glass-like appearance with no perceptible features.

^c^ Breast Imaging Reporting and Data System (BIRADS) classification:

1 Predominantly fatti breast.

2 Scattered fibroglandular densities.

3 Heterogeneously dense breast.

4 Extremely dense breast.

^d^ 5th & 95th percentiles of the differences between PD estimated with DM-Scan and Cumulus.

^e^ Concordance correlation coefficient and its 95% Con.

**Figure 3:**
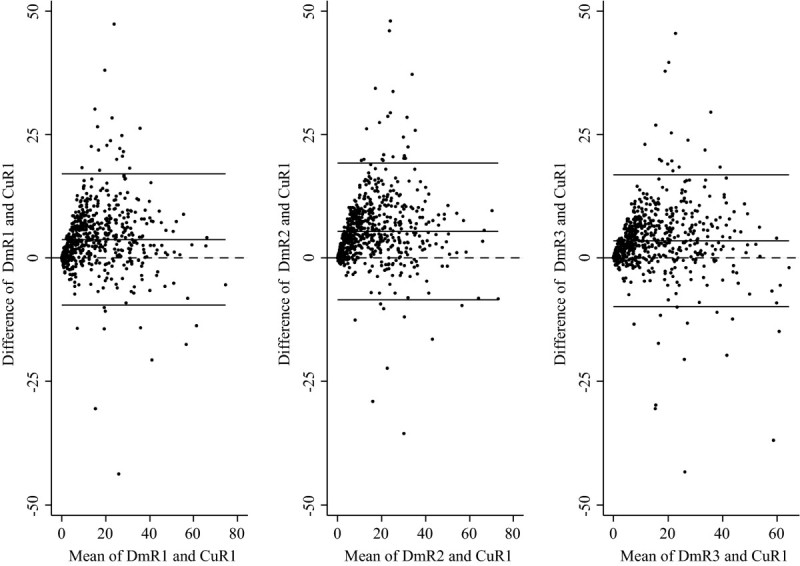
**Bland and Altman graphics comparing DM-Scan (three raters: DmR1, DmR2 & DmR3) and Cumulus (one rater, CuR1) estimates of mammographic density.**

The comparison between DM-Scan and Cumulus performance is al presented in the last row in Table 2 and Figure 3. For ninety per cent of our mammograms, PD estimates using DM-Scan were higher than those obtained using Cumulus, the mean difference ranged between 3.5% and 5.3%. In spite of this, there was substantial agreement between both tools with concordance correlation coefficients over 0.80 (0.841, 0.803 and 0.842).

### Association between several determinants of MD and PD measures using DM-scan and Cumulus

Table 3 presents the results from regression models considering PD as the dependent variable and age, menopausal status, BMI, family history of breast cancer, parity and use of hormonal replacement treatment as explanatory variables. To check the consistency of these results under different observers, a separate model was fitted for each rater. Similar results were obtained using DM-Scan and Cumulus PD estimates. A clear association of MD with age, BMI, parity and family history of breast cancer was found in all instances. Regarding menopausal status, PD tended to be higher among premenopausal women, but the differences were not statistically significant. Finally, no association was observed between use of hormonal replacement treatment (HRT) and MD. It should be noted that the number of women who were HRT users at the time of the mammogram was too small and had to be combined with ever users.

**Table 3:** **Association of age, menopausal status, BMI, family history of breast cancer, parity and use of hormonal replacement therapy with PD measures obtained with DM-Scan (3 raters) and with Cumulus (one rater)**

Variables	N	DM-Scan (Rater 1)			DM-Scan (Rater2)			DM-Scan (Rater 3)			Cumulus (Rater 1)		
		beta	95% CI^a^	***P***-value	beta	95% CI	***P***-value	beta	95% CI^a^	***P***-value	beta	95% CI^a^	***P***-value
Age													
<55	192	Ref.			Ref			Ref			Ref		
55-59	225	-3.3	-5.8 to -0.7	0.011	-4.6	-7.2 to -2.1	<0.001	-3.6	-6.1 to -1.1	0.004	-3.6	-6.1 to -1.2	0.003
>=60	221	-4.9	-7.5 to -2.3	<0.001	-5.9	-8.6 to -3.3	<0.001	-5.1	-7.7 to -2.5	<0.001	-5.2	-7.7 to -2.7	<0.001
* per 5 years*		*-2.8*	*-4.0 to -1.6*	*<0.001*	*-3.2*	*-4.5 to -2.0*	*<0.001*	*-2.7*	*-3.8 to -1.5*	*<0.001*	*-3.1*	*-4.2 to -1.9*	*<0.001*
Menopause													
Yes	570	Ref			Ref			Ref			Ref		
No	68	+1.0	-2.3 to +4.2	0.568	+1.9	-1.4 to +5.2	0.251	+0.6	-2.6 to +3.8	0.721	+0.5	-2.8 to +3.7	0.774
BMI													
<25	146	Ref.			Ref			Ref			Ref		
25-29.9	285	-7.2	-9.7 to -4.7	<0.001	-7.2	-9.7 to -4.6	<0.001	-6.5	-8.9 to -4.1	<0.001	-7.9	-10.3 to -5.5	<0.001
30-34.9	207	-14.2	-16.8 to -11.6	<0.001	-14.3	-16.9 to -11.6	<0.001	-13.4	-15.9 to -10.8	<0.001	-13.5	-16.0 to -11.0	<0.001
* per 1 unit*		*-1.1*	*-1.3 to -1.0*	<0.001	*-1.2*	*-1.4 to –1.0*	*<0.001*	*-1.1*	*-1.3 to -0.9*	*<0.001*	*-1.0*	*-1.2 to -0.9*	*<0.001*
First-degree relative with breast cancer													
No	600	Ref.			Ref			Ref			Ref		
Yes	38	+3.9	+0.0 to +7.7	0.050	+5.1	+1.2 to +9.0	0.010	+5.4	+1.6 to +9.2	0.006	+5.6	+1.9 to +9.4	0.003
Parity													
None	49	Ref			Ref			Ref			Ref		
1	78	-1.7	-6.1 to 2.6	0.439	-1.3	-5.8 to +3.1	0.552	-3.7	-8.0 to +0.6	0.093	-1.1	-5.3 to +3.3	0.616
2	297	-4.5	-8.2 to -0.8	0.018	-3.1	-6.9 to +0.7	0.109	-5.0	-8.7 to -1.4	0.007	-2.1	-5.7 to +1.5	0.244
3	167	-5.3	-9.3 to -1.4	0.008	-3.7	-7.7 to +0.3	0.069	-5.7	-9.5 to -1.8	0.004	-3.3	-7.1 to +0.5	0.092
>=4	47	-8.3	-13.2 to -3.4	0.001	-5.4	-10.3 to -0.4	0.035	-8.5	-13.3 to -3.7	0.001	-5.0	-9.7 to -0.2	0.041
per birth		*-1.9*	*-2.9 to -1.0*	*<0.001*	*-1.3*	*-2.2 to -0.3*	*0.010*	*-1.7*	*-2.7 to -0.8*	*<0.001*	*-1.2*	*-2.2 to -0.3*	*0.008*
Hormonal Replacement													
Treatment													
Never	573	Ref			Ref			Ref			Ref		
Ever (Current + Past)	7+58	+0.1	-2.9 to +3.2	0.946	+1.2	-1.9 to +4.3	0.438	+0.9	-2.1 to +3.9	0.540	+1.5	-1.5 to +4.5	0.331

^a^ 95% Confidence interval.

### Association of PD and subsequent breast cancer using DM-scan

As it has been mentioned before, the case–control study consisted of 115 cases and 119 controls. The radiologist disregarded the mammogram of a woman whose breast had previously suffered surgical reduction. Another two mammograms, both in cancer cases, were of very poor quality and the PD estimation was considered unreliable.

Table 4 displays the Odds Ratios (OR) and 95% Confidence Intervals (95% CI) of the association between PD and subsequent breast cancer development including the final set of 112 cases and 119 controls. The table also presents the association between breast cancer with menopausal status and BMI, the other two variables, apart from age, that were included in the logistic model. On average, PD estimates from DM-Scan were higher in cases than in controls (p-value=0.035). MD was categorized using as cut-offs the quartiles observed in the control population. Taking as reference a PD <7%, a PD between 17% and 28% showed a significant OR of 2.28 (95% CI: 1.03-5.04), while the highest category, PD>=29% presented an excess risk of 3.10 (95% CI: 1.35-7.14). The dose–response trend was statistically significant, with a relative linear increase in risk of 1.33 (95% CI: 1.09-1.62) per a 10% increase in PD. Neither menopausal status, nor the BMI attained statistical significance. The discriminative power of PD, adjusting for age and BMI as covariates, was 0.59 (95% CI: 0.52-0.67).

**Table 4:** **Association between BMI, menopausal status and DM-Scan estimates of density and subsequent development of breast cancer**

Variable	Controls	Cases			
	(n=119)	(n=112)	OR^a^	95% CI^a^	P-value^a^
Age: *Mean (95% CI)*	*57 (55-58 )*	*Mean 58 (57-59 )*			*0.165* ^b^
Menopausal status					
Posmenopausal	89 (75%)	83 (74%)	1.00		
Premenopausal	30 (25%)	29 (26%)	1.87	0.80-4.38	0.152
BMI					
<25	46 (39%)	40 (36%)	1.00		
25-29.9	52 (44%)	50 (45%)	1.15	0.63-2.10	0.655
>=30	21 (18%)	22 (20%)	1.39	0.61-3.12	0.429
Dm-Scan PD estimates					
*Mean (95% CI)*	*19% ( 17%-22% )*	*24% (21%-27%)*			*0.035* ^b^
<7%	30 (25%)	19 (17%)	1.00		
7%-17%	30 (25%)	21 (19%)	1.32	0.59-2.99	0.501
17%-28%	29 (24%)	32 (29%)	2.28	1.03-5.04	0.042
>=29%	30 (25%)	40 (36%)	3.10	1.35-7.14	0.008
*Per 10% increase*			*1.33*	*1.09-1.62*	*0.005*

^a^ Odds Ratio, 95% Confidence Intervals and *P*-values from a logistic.

^b^ P-value of t-test, comparing cases and controls.

^c^ Percentage of Density using DM-Scan.

## Discussion

In this study, we have presented the development and validation of a new computer-assisted tool to estimate mammographic density in full-field digital mammograms. Our results show that DM-Scan is a reliable and valid instrument to estimate MD in the context of breast cancer research. PD estimates using DM-Scan were in agreement with a visual classification of six categories (Boyd scale), and were highly concordant with those obtained using Cumulus, a similar tool developed to measure PD in digitalized film images. The reliability of PD estimations using DM-Scan is supported by the excellent agreement between the three readers in this study. Cumulus and DM-Scan estimates were equally associated with classical MD determinants, such as age, BMI, parity and family history of breast cancer. Finally, in the small case–control study designed to test the association between DM-Scan measures and breast cancer risk, ORs per category of PD showed a positive trend and, in spite of the reduced sample size, DM-Scan adjusted estimates proved to have a moderate but statistically significant discriminative power.

Our results confirm that computer-assisted tools tend to provide lower PD compared with visual assessment. This is particularly true for mammograms classified in the highest categories of density by visual inspection, where the difference between visual and computed assessment is greater than 20%. When comparing the visual evaluation of PD with the results obtained with DM-Scan and/or Cumulus, two factors may explain the wide range of variability. Firstly, a visual reading may overestimate the PD when density is higher, given that the eye evaluates the image as a whole and tends to disregard pixels or tiny regions that do not correspond to the general density pattern of the area where they are located. The second factor, previously mentioned, is the inclusion of more subcutaneous fat tissue with semi-automatic tools. This is the price we have to pay in order to obtain more reproducible results (Boyd et al. 2011;Assi et al. 2012). Regarding qualitative visual scales, they focus on particular characteristics of the mammographic tissue and do not directly measure PD. Different studies have reported large differences between MD assessment based on qualitative and quantitative methods (Yaffe 2008). The lack of a perfect agreement between PD and mammographic patterns considered by qualitative scales has been interpreted as a proof of the existence of additional information in the mammogram, observable by radiologists, that is not captured by PD alone (Manduca et al. 2009). In fact, recent papers have emphasized the importance of textural information relating breast images with breast cancer risk, but the biological significance of these characteristics is still unknown (Li et al. 2012;Nielsen et al. 2011). For the time being, quantitative methods provide more precise and reliable measures and are less influenced by subjectivity (Yaffe 2008).

DM-Scan PD estimates were highly reproducible, according to our study. In fact, even though our set of radiologists showed good inter-rater agreement in the visual classification of PD using Boyd semi-quantitative scale, concordance estimates were lower than those found here using DM-Scan (Perez-Gomez et al. 2012). Unfortunately, we could not compare the reproducibility of PD measures using Cumulus, since only one of our radiologists had used it before. However, it should be noted that she considered DM-Scan more user-friendly, probably due to the fact that DM-Scan automatically delineates the breast contour and the oblique muscle, providing that the mammogram has a reasonable quality. DM-Scan also offers a preliminary assessment of PD that can be modified by the user if he/she considers it inadequate. Regarding the comparison of DM-Scan with Cumulus, there was a good agreement in the estimates obtained using these two tools, but it is interesting to note that, on average, DM-Scan estimations were higher than those obtained with Cumulus (a difference in PD between 4% and 5%). This result should be confirmed by other studies.

Our results show that DM-Scan and Cumulus seem to capture the same overall associations with risk factors for breast cancer (i.e. age, BMI and reproductive factors). However, we failed to find an association between PD and menopausal status with any of these tools, probably due to the small number of premenopausal women. Regarding the use of hormonal replacement therapy, the number of current users was insufficient to analyze them as a single category, but MD was not higher in this group of women in the DDM-Spain study, and most of these women were under estrogen-only therapy (Pollan et al. 2012).

Our small case–control study served to confirm that DM-Scan estimates are related with breast cancer risk: PD was higher in cases than in controls and a clear dose–response relationship was observed in the association between PD and subsequent breast cancer. The discriminative power found here was similar to that reported in other studies (Manduca et al. 2009;Vachon et al. 2007).

Among the technical advantages of DM-Scan it is worth mentioning its availability for both Windows and Linux O.S., and its independence of proprietary software. Also, in contrast to Cumulus, DM-Scan presents, among other features, a more user-friendly interface, especially in relation to the procedure of defining the batch of images to be analysed, and a breast filter (as explained in the Material and Methods section) which allows for a better recognition of dense tissue. A full version of DM-Scan is freely available under request.

We would like to highlight several strengths of this study: DDM-Spain is a population-based study, with information regarding breast cancer risk factors collected in a homogeneous way by trained interviewers who also measured weight and height under the same protocol using the same tools (Pollan et al. 2012). The three readers were experienced radiologists and were blinded to the risk factors. Finally, the case–control study was also population-based, among attendants to a screening center, Burjassot, with extensive experience using full-field mammograms. PD assessment was also performed in a blind way, mixing case and control images.

Our study also has several limitations. Firstly, DM-Scan and Cumulus were used on processed mammograms that depend on the manufacturers. We did not have access to unprocessed (raw) images because Spanish screening centers discard them due to storage constraints. However, recent studies have confirmed that density measures in raw and processed images are strongly correlated, have equal reliability and are similarly associated with breast cancer (Keller et al. 2013;Vachon et al. 2013). In the same way, a recent paper has shown that image acquisition parameters not available here, such as compressed breast thickness, compression force and others, do not modify the association between PD and breast cancer risk (Olson et al. 2012). Secondly, even though our purpose was to study DM-Scan validity and reliability, it would have been interesting to compare the reliability using DM-Scan and Cumulus, something we could not achieve here. Nevertheless, the high inter-rater concordance obtained with DM-Scan confirms that the new tool is, at least, equally valid to obtain reliable results. Thirdly, the case–control study had limited power and the information regarding BMI was self-reported. The size was too small to consider more categories of density and to explore the stability of the association between PD estimates and subsequent breast cancer in subgroups of women. In spite of these constraints, our results were equivalent to those obtained in larger datasets using Cumulus or other tools (Vachon et al. 2007;McCormack & dos Santos Silva 2006;Stone et al. 2010;Yaghjyan et al. 2011). Finally, even though DM-Scan has a friendly interface and is relatively easy to use, the user still has to remove unwanted characteristics in the mammogram and manipulate the software to establish what he/she believes represents the right amount of dense tissue. A fully-automated version based on machine learning techniques is currently under development and will be available in the short term.

In conclusion, MD measures obtained with DM-are highly reproducible and show the expected association with those factors that influence breast. Moreover, DM-Scan estimates among women who subsequently developed breast cancer were higher than those obtained in health controls of the same age. These results demonstrate that DM-Scan is a valid and reliable tool to assess mammographic density in full-field digital images.

## Electronic supplementary material

Additional file 1: Bland and Altman graphics comparing DM-Scan estimates obtained by three different readers (DmR1, DmR2 & DmR3). (PPT 255 KB)

Below are the links to the authors’ original submitted files for images.

Authors’ original file for figure 1

Authors’ original file for figure 2

Authors’ original file for figure 3

## Abbreviations

BC: Breast cancer
BMI: Body mass index
DT: Dense tissue
FT: Fat tissue
HRT: Hormonal replacement treatment
MD: Mammographic density
OR: Odds ratio
PD: Percent of density
95%CI: 95% Confidence intervals.

## References

[CR1] Ascunce N, Salas D, Zubizarreta R, Almazan R, Ibanez J, Ederra M (2010). Cancer screening in Spain. Ann Oncol.

[CR2] Assi V, Warwick J, Cuzick J, Duffy SW (2012). Clinical and epidemiological issues in mammographic density. Nat Rev Clin Oncol.

[CR3] Bland JM, Altman DG (1986). Statistical methods for assessing agreement between two methods of clinical measurement. Lancet.

[CR4] Boyd NF, Martin LJ, Yaffe MJ, Minkin S (2011). Mammographic density and breast cancer risk: current understanding and future prospects. Breast Cancer Res.

[CR5] Byng JW, Yaffe MJ, Jong RA, Shumak RS, Lockwood GA, Tritchler DL, Boyd NF (1998). Analysis of mammographic density and breast cancer risk from digitized mammograms. Radiographics.

[CR6] Cabanes A, Pastor-Barriuso R, Garcia-Lopez M, Pedraz-Pingarron C, Sanchez-Contador C, Vazquez Carrete JA, Moreno MP, Vidal C, Salas D, Miranda-Garcia J (2011). Alcohol, tobacco, and mammographic density: a population-based study. Breast Cancer Res Treat.

[CR7] Cuzick J, Warwick J, Pinney E, Duffy SW, Cawthorn S, Howell A, Forbes JF, Warren RM (2011). Tamoxifen-induced reduction in mammographic density and breast cancer risk reduction: a nested case–control study. J Natl Cancer Inst.

[CR8] Evans DG, Warwick J, Astley SM, Stavrinos P, Sahin S, Ingham S, McBurney H, Eckersley B, Harvie M, Wilson M (2012). Assessing individual breast cancer risk within the U.K. National Health Service Breast Screening Program: a new paradigm for cancer prevention. Cancer Prev Res (Phila).

[CR9] Garrido-Estepa M, Ruiz-Perales F, Miranda J, Ascunce N, Gonzalez-Roman I, Sanchez-Contador C, Santamarina C, Moreo P, Vidal C, Peris M (2010). Evaluation of mammographic density patterns: reproducibility and concordance among scales. BMC Cancer.

[CR10] Harvey JA (2004). Quantitative assessment of percent breast density: analog versus digital acquisition. Technol Cancer Res Treat.

[CR11] Highnam RP, Brady JM, Shepstone BJ (1998). Estimation of compressed breast thickness during mammography. Br J Radiol.

[CR12] Keller BM, Nathan DL, Gavenonis SC, Chen J, Conant EF, Kontos D (2013). Reader variability in breast density estimation from full-field digital mammograms: the effect of image postprocessing on relative and absolute measures. Acad Radiol.

[CR13] Li J, Szekely L, Eriksson L, Heddson B, Sundbom A, Czene K, Hall P, Humphreys K (2012). High-throughput mammographic-density measurement: a tool for risk prediction of breast cancer. Breast Cancer Res.

[CR14] Lin LI (1989). A concordance correlation coefficient to evaluate reproducibility. Biometrics.

[CR15] Manduca A, Carston MJ, Heine JJ, Scott CG, Pankratz VS, Brandt KR, Sellers TA, Vachon CM, Cerhan JR (2009). Texture features from mammographic images and risk of breast cancer. Cancer Epidemiol Biomarkers Prev.

[CR16] McCormack VA, dos Santos Silva I (2006). Breast density and parenchymal patterns as markers of breast cancer risk: a meta-analysis. Cancer Epidemiol Biomarkers Prev.

[CR17] Nielsen M, Karemore G, Loog M, Raundahl J, Karssemeijer N, Otten JD, Karsdal MA, Vachon CM, Christiansen C (2011). A novel and automatic mammographic texture resemblance marker is an independent risk factor for breast cancer. Cancer Epidemiol.

[CR18] Olson JE, Sellers TA, Scott CG, Schueler BA, Brandt KR, Serie DJ, Jensen MR, Wu FF, Morton MJ, Heine JJ (2012). The influence of mammogram acquisition on the mammographic density and breast cancer association in the mayo mammography health study cohort. Breast Cancer Res.

[CR19] Perez-Gomez B, Ruiz F, Martinez I, Casals M, Miranda J, Sanchez-Contador C, Vidal C, Llobet R, Pollan M, Salas D (2012). Women's features and inter-/intra-rater agreement on mammographic density assessment in full-field digital mammograms (DDM-SPAIN). Breast Cancer Res Treat.

[CR20] Pollan M, Lope V, Miranda-Garcia J, Garcia M, Casanova F, Sanchez-Contador C, Santamarina C, Moreo P, Vidal C, Peris M (2012). Adult weight gain, fat distribution and mammographic density in Spanish pre- and post-menopausal women (DDM-Spain). Breast Cancer Res Treat.

[CR21] Sala M, Salas D, Belvis F, Sanchez M, Ferrer J, Ibanez J, Roman R, Ferrer F, Vega A, Laso MS (2011). Reduction in false-positive results after introduction of digital mammography: analysis from four population-based breast cancer screening programs in Spain. Radiology.

[CR22] Schousboe JT, Kerlikowske K, Loh A, Cummings SR (2011). Personalizing mammography by breast density and other risk factors for breast cancer: analysis of health benefits and cost-effectiveness. Ann Intern Med.

[CR23] Stone J, Ding J, Warren RM, Duffy SW, Hopper JL (2010). Using mammographic density to predict breast cancer risk: dense area or percentage dense area. Breast Cancer Res.

[CR24] Vachon CM, Brandt KR, Ghosh K, Scott CG, Maloney SD, Carston MJ, Pankratz VS, Sellers TA (2007). Mammographic breast density as a general marker of breast cancer risk. Cancer Epidemiol Biomarkers Prev.

[CR25] Vachon C, Fowler EE, Tiffenberg G, Scott C, Pankratz VS, Sellers TA, Heine JJ (2013). Comparison of percent density from raw and processed full field digital mammography data. Breast Cancer Res.

[CR26] Yaffe MJ (2008). Mammographic density. Measurement of mammographic density. Breast Cancer Res.

[CR27] Yaghjyan L, Colditz GA, Collins LC, Schnitt SJ, Rosner B, Vachon C, Tamimi RM (2011). Mammographic breast density and subsequent risk of breast cancer in postmenopausal women according to tumor characteristics. J Natl Cancer Inst.

