# Supplementary material for: Validation of DM-Scan, a computer-assisted tool to assess mammographic density in full-field digital mammograms
Source: Springerplus. 2013 May 24;2(1):242. doi: 10.1186/2193-1801-2-242 (PMC3693435; doi:10.1186/2193-1801-2-242)
Supplement: Supplementary file 1 — Additional file 1: Bland and Altman graphics comparing DM-Scan estimates obtained by three different readers (DmR1, DmR2 & DmR3). (PPT 255 KB) [file 40064_2013_333_MOESM1_ESM.ppt]

## Slide 1
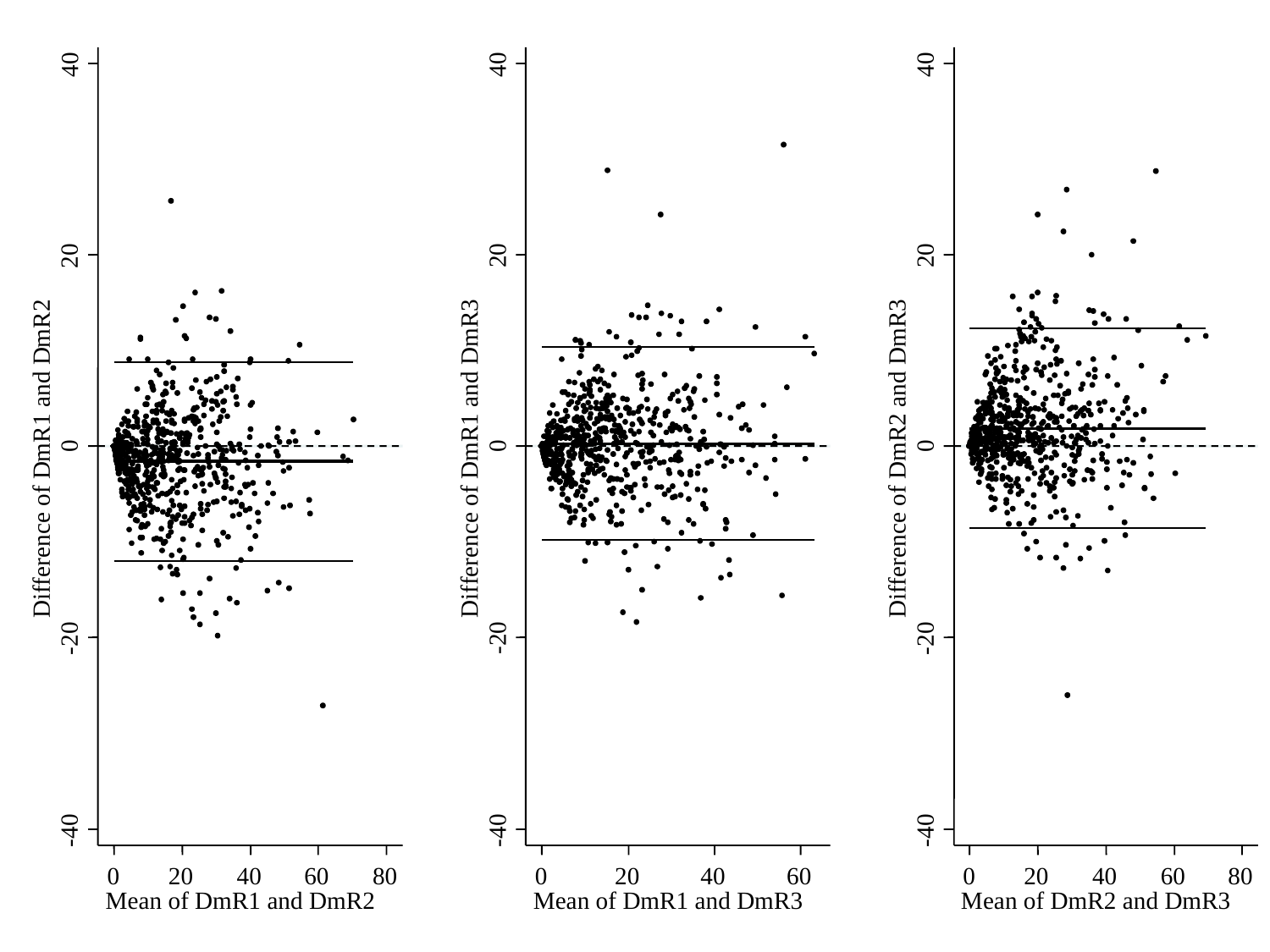

40
40
40
20
20
20
0
0
0
Difference of DmR1 and DmR3
Difference of DmR2 and DmR3
Difference of DmR1 and DmR2
-20
-20
-20
-40
-40
-40
0
20
40
60
80
0
20
40
60
0
20
40
60
80
Mean of DmR1 and DmR2
Mean of DmR1 and DmR3
Mean of DmR2 and DmR3
