# Supplementary figures and images for: Validation of DM-Scan, a computer-assisted tool to assess mammographic density in full-field digital mammograms
Source: Springerplus. 2013 May 24;2(1):242. doi: 10.1186/2193-1801-2-242 (PMC3693435; doi:10.1186/2193-1801-2-242)

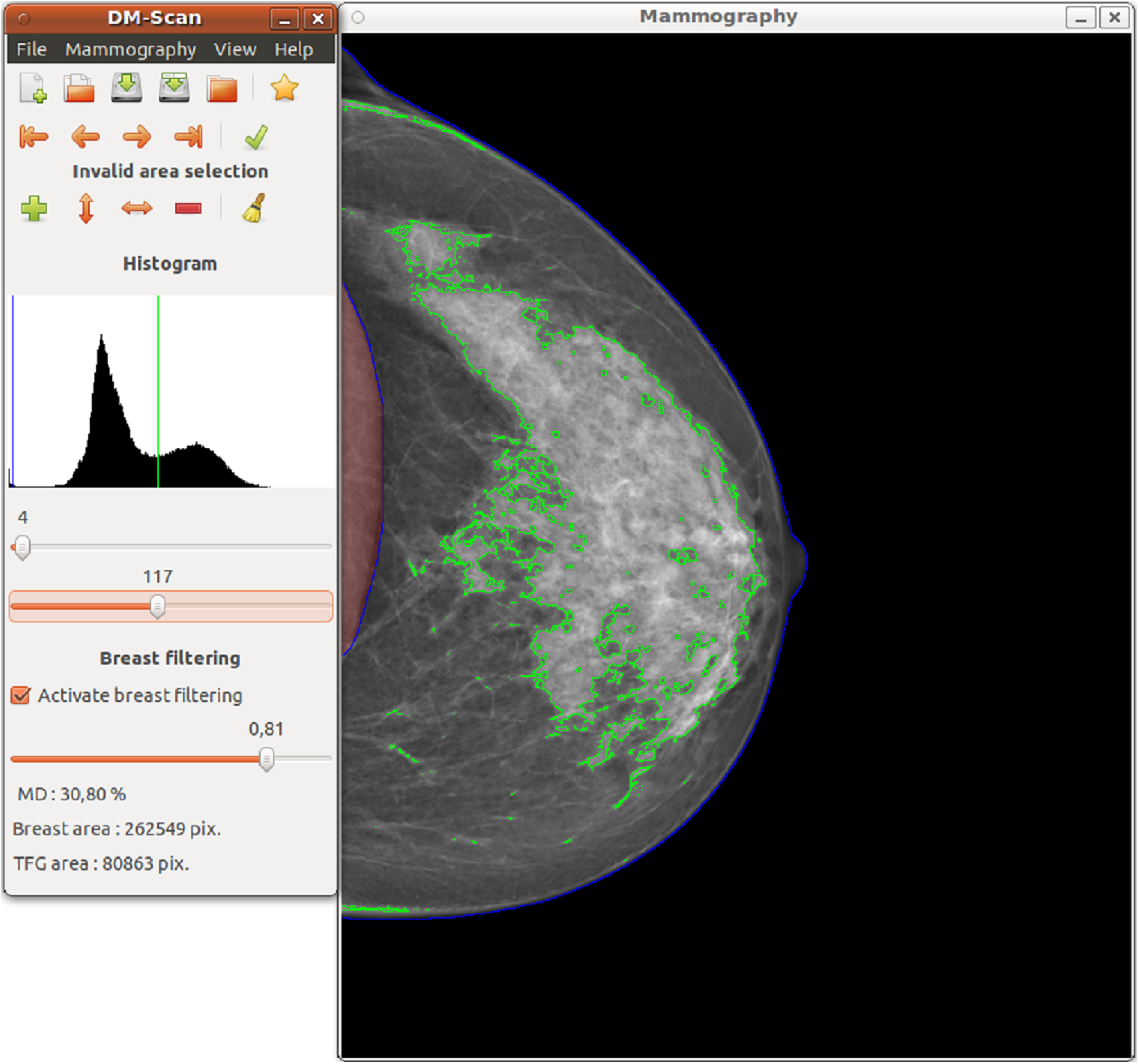

Supplement: Supplementary file 2 — Authors’ original file for figure 1 [file 40064_2013_333_MOESM2_ESM.tiff]

DM-scan & Cumulus PD estimates

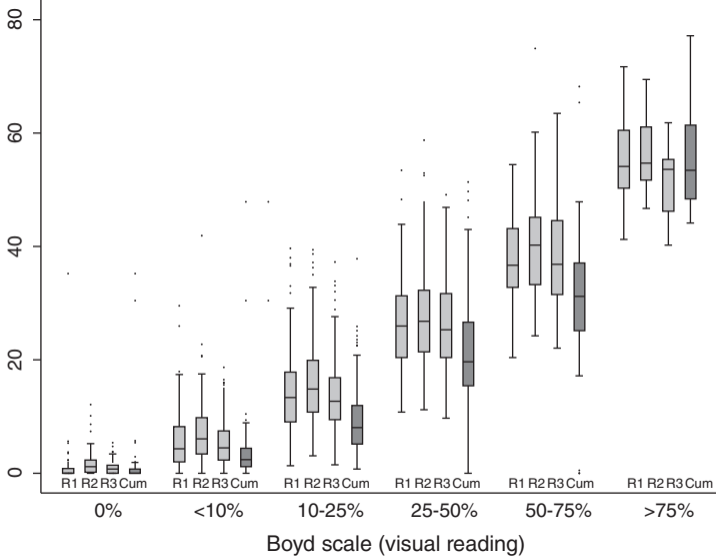

Supplement: Supplementary file 3 — Authors’ original file for figure 2 [file 40064_2013_333_MOESM3_ESM.pdf]

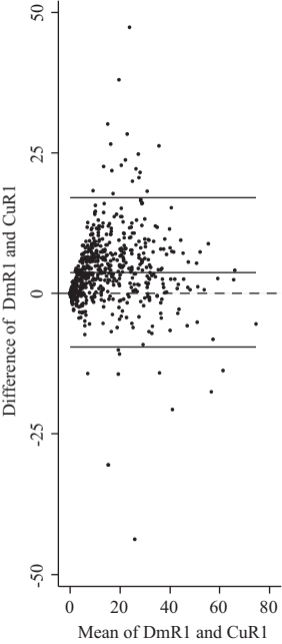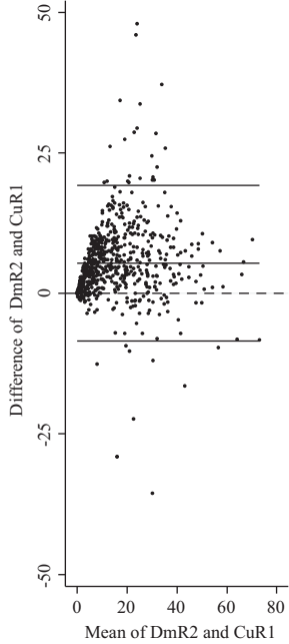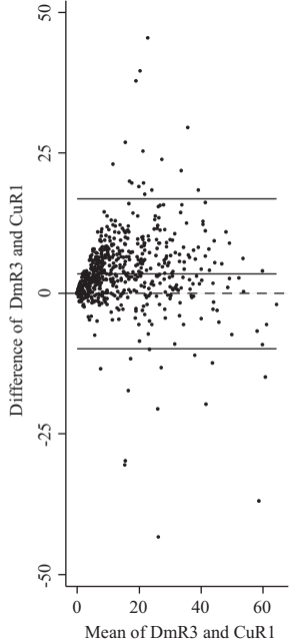

Supplement: Supplementary file 4 — Authors’ original file for figure 3 [file 40064_2013_333_MOESM4_ESM.pdf]
